# Data Flow Analysis of Asynchronous Systems using Infinite Abstract Domains

**DOI:** 10.1007/978-3-030-72019-3_2

**Published:** 2021-03-23

**Authors:** Snigdha Athaiya, Raghavan Komondoor, K. Narayan Kumar

**Affiliations:** Imperial College, London, UK; 9 Indian Institute of Science, Bengaluru, India; 10 Chennai Mathematical Institute, Chennai, India

**Keywords:** Data Flow Analysis, Message-passing systems

## Abstract

Asynchronous message-passing systems are employed frequently to implement distributed mechanisms, protocols, and processes. This paper addresses the problem of precise data flow analysis for such systems. To obtain good precision, data flow analysis needs to somehow skip execution paths that read more messages than the number of messages sent so far in the path, as such paths are infeasible at run time. Existing data flow analysis techniques do elide a subset of such infeasible paths, but have the restriction that they admit only finite abstract analysis domains. In this paper we propose a generalization of these approaches to admit infinite abstract analysis domains, as such domains are commonly used in practice to obtain high precision. We have implemented our approach, and have analyzed its performance on a set of 14 benchmarks. On these benchmarks our tool obtains significantly higher precision compared to a baseline approach that does not elide any infeasible paths and to another baseline that elides infeasible paths but admits only finite abstract domains.

### 1 Introduction

Distributed software that communicates by asynchronous message passing is a very important software paradigm in today's world. It is employed in varied domains, such as distributed protocols and workflows, event-driven systems, and UI-based systems. Popular languages used in this domain include Go (https://golang.org/), Akka (https://akka.io/), and P (https://github.com/p-org).

Analysis and verification of asynchronous systems is an important problem, and poses a rich set of challenges. The research community has focused historically on a variety of approaches to tackle this overall problem, such as model checking and systematic concurrency testing [25,13], formal verification to check properties such as reachability or coverability of states [41,3,2,21,18,31,19,1], and data flow analysis [29].

Data flow analysis [32,30] is a specific type of verification technique that propagates values from an *abstract domain* while accounting for all paths in a program. It can hence be used to check whether a property or assertion always holds. The existing verification and data flow analysis approaches mentioned earlier have a major limitation, which is that they admit only finite abstract domains. This, in general, limits the classes of properties that can be successfully verified. On the other hand, data flow analysis of sequential programs using infinite abstract domains, e.g., *constant propagation* [32], *interval analysis* [12], and *octagons* [44], is a well developed area, and is routinely employed in verification settings. In this paper we seek to bridge this fundamental gap, and develop a precise data flow analysis framework for message-passing asynchronous systems that admits infinite abstract domains.

#### 1.1 Motivating Example: Leader election

$max$ := process number; send $\langle 1, max \rangle$
Process is in *active* mode
**while** true **do**
    **if** process is in *passive* mode **then**
        receive a mesg and send this same mesg
    **else if** message $\langle 1, i \rangle$ arrives **then**
        **if** $i \neq max$ **then**
            Send message $\langle 2, i \rangle$; $left := i$
        **else**
            Declare $max$ as the global maximum
            nr_leaders++; assert(nr_leaders = 1)
    **else if** message $\langle 2, j \rangle$ arrives **then**
        **if** $left > j$ and $left > max$ **then**
            $max := left$
            Send message $\langle 1, max \rangle$
        **else**
            Process enters *passive* mode

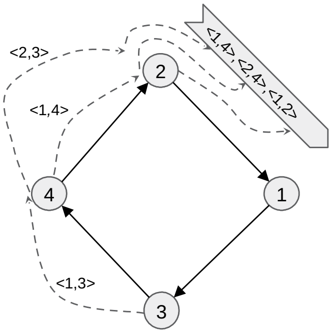

**Fig. 1.** Pseudo-code of each process in leader election, and a partial run

To motivate our work we use a benchmark program[3] in the *Promela* language [25] that implements a *leader election* protocol [17]. In the protocol there is a ring of processes, and each process has a unique number. The objective is to discover the "leader", which is the process with the maximum number. The pseudo-code of each process in the protocol is shown in the left side of Figure 1. Each process has its own copy of local variables *max* and *left*, whereas nr_leaders is a global variable that is common to all the processes (its initial value is zero). Each process sends messages to the next process in the ring via an unbounded FIFO channel. Each process becomes "ready" whenever a message is available for it to receive, and at any step of the protocol any one ready process (chosen

[3] file `assertion.leader.prm` in www.imm.dtu.dk/~albl/promela-models.zip.

non-deterministically) executes one iteration of its “while” loop. (We formalize these execution rules in a more general fashion in Section 2.1.) The messages are a 2-tuple $\langle x, i\rangle$, where $x$ can be 1 or 2, and $1 \leq i \leq max$. The right side of Figure 1 shows a snapshot at an intermediate point during a run of the protocol. Each dashed arrow between two nodes represents a send of a message and a (completed) receipt of the same message. The block arrow depicts the channel from Process 2 to Process 1, which happens to contain three sent (but still unreceived) messages.

It is notable that in any run of the protocol, Lines 10-11 happen to get executed only by the actual leader process, and that too, exactly once. Hence, the assertion never fails. The argument for this claim is not straightforward, and we refer the reader to the paper [17] for the details.

#### 1.2 Challenges in property checking

Data flow analysis could be used to verify the assertion in the example above, e.g., using the *Constant Propagation* (CP) abstract domain. This analysis determines at each program point whether each variable has a fixed value, and if yes, the value itself, across all runs that reach the point. In the example in Figure 1, all actual runs of the system that happen to reach Line 10 come there with value zero for the global variable nr_leaders.

A challenge for data flow analysis on message-passing systems is that there may exist *infeasible* paths in the system. These are paths with more receives of a certain message than the number of copies of this message that have been sent so far. For instance, consider the path that consists of two back-to-back iterations of the “while” loop by the leader process, both times through Lines 3,6,9-11. This path is not feasible, due to the impossibility of having two copies of the message $\langle 1, max\rangle$ in the input channel [17]. The second iteration would bring the value 1 for nr_leaders at Line 10, thus inferring a non-constant value and hence declaring the assertion as failing (which would be a false positive).

Hence, it is imperative in the interest of precision for any data flow analysis or verification approach to track the channel contents as part of the exploration of the state space. Tracking the contents of unbounded channels precisely is known to be undecidable even when solving problems such as reachability and coverability (which are simpler than data flow analysis). Hence, existing approaches either bound the channels (which in general causes unsoundness), or use sound abstractions such as *unordered channels* (also known as the Petri Net or VASS abstraction) or *lossy channels*. Such abstractions suffice to elide a subset of infeasible paths. In our running example, the unordered channel abstraction happens to suffice to elide infeasible paths that could contribute to a false positive at the point of the assertion. However, the analysis would need to use an abstract domain such as CP to track the values of integer variables. This is an infinite domain (due to the infinite number of integers). The most closely related previous dataflow analysis approach for distributed systems [29] does use the unordered channel abstraction, but does not admit infinite abstract domains, and hence cannot verify assertions such as the one in the example above.

#### 1.3 Our Contributions

This paper is the first one to the best of our knowledge to propose an approach for data flow analysis for asynchronous message-passing systems that (a) admits infinite abstract domains, (b) uses a reasonably precise channel abstraction among the ones known in the literature (namely, the unordered channels abstraction), and (c) computes maximally precise results possible under the selected channel abstraction. Every other approach we are aware of exhibits a strict subset of the three attributes listed above. It is notable that previous approaches do tackle the infinite state space induced by the unbounded channel contents. However, they either do not reason about variable values at all, or only allow variables that are based on finite domains.

Our primary contribution is an approach that we call *Backward DFAS*. This approach is maximally precise, and admits a class of infinite abstract domains. This class includes well-known examples such as Linear Constant Propagation (LCP) [51] and Affine Relationships Analysis (ARA) [46], but does not include the full (CP) analysis. We also propose another approach, which we call *Forward DFAS*, which admits a broader class of abstract domains, but is not guaranteed to be maximally precise on all programs.

We describe a prototype implementation of both our approaches. On a set of 14 real benchmarks, which are small but involve many complex idioms and paths, our tool verifies approximately 50% more assertions than our implementation of the baseline approach [29].

The rest of the paper is structured as follows. Section 2 covers the background and notation that will be assumed throughout the paper. We present the Backward DFAS approach in Section 3, and the Forward DFAS approach in Section 4. Section 5 discusses our implementation and evaluation. Section 6 discusses related work, and Section 7 concludes the paper.

### 2 Background and Terminology

Vector addition systems with states or VASS [27] are a popular modelling technique for distributed systems. We begin this section by defining an extension to VASS, which we call a *VASS-Control Flow Graph* or *VCFG*.

**Definition 1.** *A* VASS-Control Flow Graph *or VCFG $\mathcal{G}$ is a graph, and is described by the tuple $\langle Q, \delta, r, q_0, V, \pi, \theta \rangle$, where*
*$Q$ is a finite set of nodes, $\delta \subseteq Q \times Q$ is a finite set of edges,*
*$r \in \mathbb{N}$, $q_0$ is the* start *node, $V$ is a set of variables or memory locations,*
*$\pi : \delta \to A$ maps each edge to an* action, *where $A \equiv ((V \to \mathbb{Z}) \to (V \to \mathbb{Z}))$,*
*$\theta : \delta \to \mathbb{Z}^r$ maps each edge to a vector in $\mathbb{Z}^r$.*

For any edge $e = (q_1, q_2) \in \delta$, if $\pi(e) = a$ and $\theta(e) = w$, then $a$ is called the *action* of $e$ and $w$ is called the *queuing vector* of $e$. This edge is depicted as $q_1 \xrightarrow{a,w} q_2$. The variables and the *actions* are the only additional features of a VCFG over VASS.

A *configuration* of a VCFG is a tuple $\langle q, c, \xi \rangle$, where $q \in Q$, $c \in \mathbb{N}^r$ and $\xi \in (V \to \mathbb{Z})$. The initial configuration of a VCFG is $\langle q_0, \mathbf{0}, \xi_0 \rangle$, where $\mathbf{0}$ denotes a vector with $r$ zeroes, and $\xi_0$ is a given initial valuation for the variables. The VCFG can be said to have $r$ *counters*. The vector $c$ in each configuration can be thought of as a valuation to the counters. The transitions between VCFG configurations are according to the rule below:

$$\frac{e = (q_1, q_2),\ e \in \delta,\ \pi(e) = a,\ \theta(e) = w,\ a(\xi_1) = \xi_2,\ c_1 + w = c_2,\ c_2 \geq \mathbf{0}}{\langle q_1, c_1, \xi_1 \rangle \Rightarrow_e \langle q_2, c_2, \xi_2 \rangle}$$

#### 2.1 Modeling of Asynchronous Message Passing Systems as VCFGs

Asynchronous systems are composed of finite number of independently executing processes that communicate with each other by passing messages along FIFO channels. The processes may have local variables, and there may exist shared (or global) variables as well. For simplicity of presentation we assume all variables are global.

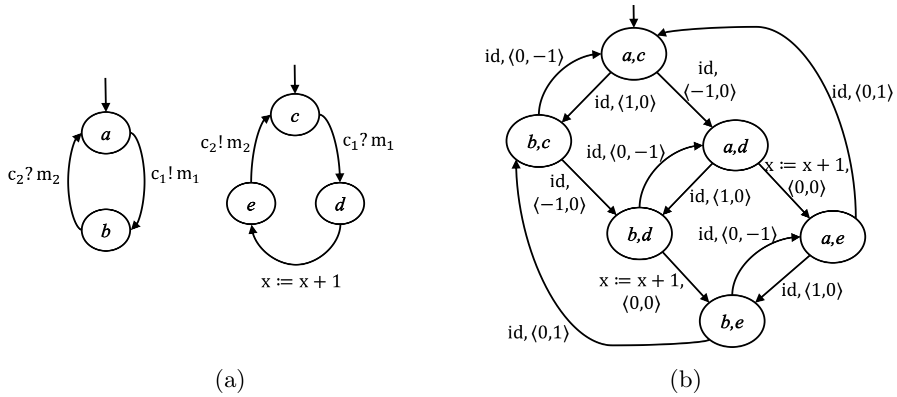

**Fig. 2.** (a) Asynchronous system with two processes, (b) its VCFG model

Figure 2(a) shows a simple asynchronous system with two processes. In this system there are two channels, $c_1$ and $c_2$, and a message alphabet consisting of two elements, $m_1$ and $m_2$. The semantics we assume for message-passing systems is the same as what is used by the tool Spin [25]. A configuration of the system consists of the current control states of all the processes, the contents of all the channels, and the values of all the variables. A single transition of the system consists of a transition of one of the processes from its current control-state to a successor control state, accompanied with the corresponding queuing operation or variable-update action. A transition labeled c ! m can be taken unconditionally, and results in 'm' being appended to the tail of the channel 'c'. A transition labeled c ? m can be taken only if an instance of 'm' is available at the head of ‘c’, and results in this instance getting removed from ‘c’. (Note, based on the context, we over-load the term “message” to mean either an element of the message alphabet, or an instance of a message-alphabet element in a channel at run-time.)

Asynchronous systems can be modeled as VCFGs, and our approach performs data flow analysis on VCFGs. We now illustrate how an asynchronous system can be modeled as a VCFG. We assume a fixed number of processes in the system. We do this illustration using the example VCFG in Figure 2(b), which models the system in Figure 2(a). Each node of the VCFG represents a tuple of control-states of the processes, while each edge corresponds to a transition of the system. The action of a VCFG edge is identical to the action that labels the corresponding process transition. (“id” in Figure 2(b) represents the *identity* action) The VCFG will have as many counters as the number of unique pairs $(\mathrm{c_i}, \mathrm{m_j})$ such that the operation $\mathrm{c_i} \,!\, \mathrm{m_j}$ is performed by any process. If an edge $e$ in the VCFG corresponds to a send transition $\mathrm{c_i} \,!\, \mathrm{m_j}$ of the system, then $e$’s queuing vector would have a +1 for the counter corresponding to $(\mathrm{c_i}, \mathrm{m_j})$ and a zero for all the other counters. Analogously, a receive operation gets modeled as -1 in the queuing vector. In Figure 2(b), the first counter is for $(\mathrm{c_1},\mathrm{m_1})$ while the second counter is for $(\mathrm{c_2},\mathrm{m_2})$. Note that the +1 and -1 encoding (which are inherited from VASS’s) effectively cause FIFO channels to be treated as unordered channels.

When each process can invoke procedures as part of its execution, such systems can be modeled using *inter-procedural* VCFGs, or iVCFGs. These are extensions of VCFGs just as standard inter-procedural control-flow graphs are extensions of control-flow graphs. Constructing an iVCFG for a given system is straightforward, under a restriction that at most one of the processes in the system can be executing a procedure other than its main procedure at any time. This restriction is also present in other related work [29,5].

#### 2.2 Data flow analysis over iVCFGs

Data flow analysis is based on a given *complete lattice* $\mathcal{L}$, which serves as the abstract domain. As a pre-requisite step before we can perform our data flow analysis on iVCFGs, we first consider each edge $v \xrightarrow{a,w} w$ in each procedure in the iVCFG, and replace the (concrete) action $a$ with an abstract action $f$, where $f : \mathcal{L} \to \mathcal{L}$ is a given abstract transfer function that *conservatively over-approximates* [12] the behavior of the concrete action $a$.

Let $p$ be a path in a iVCFG, let $p_0$ be the first node in the path, and let $\xi_i$ be a valuation to the variables at the beginning of $p$. The path $p$ is said to be *feasible* if, starting from the configuration $\langle p_0, \mathbf{0}, \xi_i \rangle$, the configuration $\langle q, d, \xi \rangle$ obtained at each successive point in the path is such that $d \geq \mathbf{0}$, with successive configurations along the path being generated as per the rule for transitions among VCFG configurations that was given before Section 2.1. For any path $p = e_1\, e_2 \ldots e_k$ of an iVCFG, we define its *path transfer function* $\mathit{ptf}(p)$ as $f_{e_k} \circ f_{e_{k-1}} \ldots \circ f_{e_1}$, where $f_e$ is the abstract action associated with edge $e$.

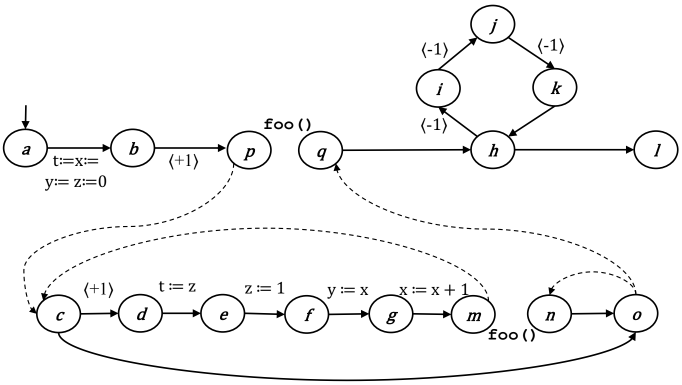

**Fig. 3.** Example iVCFG

The standard data flow analysis problem for sequential programs is to compute the join-over-all-paths (JOP) solution. Our problem statement is to compute the join-over-all-feasible-paths (JOFP) solution for iVCFGs. Formally stated, if *start* is the entry node of the "main" procedure of the iVCFG, given any node *target* in any procedure of the iVCFG, and an "entry" value $d_0 \in \mathcal{L}$ at *start* such that $d_0$ conservatively over-approximates $\xi_0$, we wish to compute the JOFP value at *target* as defined by the following expression:

$$\bigsqcup_{\substack{p \text{ is a feasible and interprocedurally valid} \\ \text{path in the iVCFG from } start \text{ to } target}} (ptf(p))(d_0)$$

Intuitively, due to the unordered channel abstraction, every run of the system corresponds to a feasible path in the iVCFG, but not vice versa. Hence, the JOFP solution above is guaranteed to conservatively over-approximate the JOP solution on the *runs* of the system (which is not computable in general).

### 3 Backward DFAS Approach

In this section we present our key contribution – the *Backward DFAS* (Data Flow Analysis of Asynchronous Systems) algorithm – an interprocedural algorithm that computes the precise JOFP at any given node of the iVCFG.

We begin by presenting a running example, which is the iVCFG with two procedures depicted in Figure 3. There is only one channel and one message in the message alphabet in this example, and hence the *queuing vectors* associated with the edges are of size 1. The edges without the vectors are implicitly associated with zero vectors. The *actions* associated with edges are represented in the form of assignment statements. The edges without assignment statements next to them have *identity* actions. The upper part of the Figure 3, consisting of nodes

$a, b, p, q, h, i, j, k, l$, is the VCFG of the "main" procedure. The remaining nodes constitute the VCFG of the (tail) recursive procedure `foo`. The solid edges are intra-procedural edges, while dashed edges are inter-procedural edges.

Throughout this section we use *Linear Constant Propagation* (LCP) [51] as our example data flow analysis. LCP, like CP, aims to identify the variables that have constant values at any given location in the system. LCP is based on the same infinite domain as CP; i.e., each abstract domain element is a mapping from variables to (integer) values. The "$\sqsupseteq$" relation for the LCP lattice is also defined in the same way as for CP. The encoding of the transfer functions in LCP is as follows. Each edge (resp. path) maps the outgoing value of each variable to *either* a constant, *or* to a linear expression in the incoming value of at most one variable into the edge (resp. path), *or* to a special symbol $\top$ that indicates an unknown outgoing value. For instance, for the edge $g \to m$ in Figure 3, its transfer function can be represented symbolically as (t'=t,x'=x+1,y'=y,z'=z), where the primed versions represent outgoing values and unprimed versions represent incoming values.

Say we wish to compute the JOFP at node $k$. The only feasible paths that reach node $k$ are the ones that attain calling-depth of three or more in the procedure `foo`, and hence encounter at least three *send* operations, which are required to clear the three *receive* operations encountered from node $h$ to node $k$. All such paths happen to bring the constant values (t = 1, z = 1) to the node $k$. Hence, (t = 1, z = 1) is the precise JOFP result at node $k$. However, infeasible paths, if not elided, can introduce imprecision. For instance, the path that directly goes from node $c$ to node $o$ in the outermost call to the Procedure `foo` (this path is of calling-depth zero) brings values of zero for all four variables, and would hence prevent the precise fact (t = 1, z = 1) from being inferred.

#### 3.1 Assumptions and Definitions

The set of all $\mathcal{L} \to \mathcal{L}$ transfer functions clearly forms a complete lattice based on the following ordering: $f_1 \sqsupseteq f_2$ iff for all $d \in \mathcal{L}$, $f_1(d) \sqsupseteq f_2(d)$. Backward DFAS makes a few assumptions on this lattice of transfer functions. The first is that this lattice be of *finite height*; i.e., all strictly ascending chains of elements in this lattice are finite (although no a priori bound on the sizes of these chains is required). The second is that a representation of transfer functions is available, as are operators to compose, join, and compare transfer functions. Note, the two assumptions above are also made by the classical "functional" inter-procedural approach of Sharir and Pnueli [55]. Thirdly, we need distributivity, as defined below: for any $f_1, f_2, f \in \mathcal{L} \to \mathcal{L}$, $(f_1 \sqcup f_2) \circ f = (f_1 \circ f) \sqcup (f_2 \circ f)$. The distributivity assumption is required only if the given system contains recursive procedure calls.

Linear Constant Propagation (LCP) [51] and Affine Relationships Analysis (ARA) [46] are well-known examples of analyses based on infinite abstract domains that satisfy all of the assumptions listed above. Note that the CP transfer-functions lattice is not of finite height. Despite the LCP abstract domain being the same as the CP abstract domain, the encoding chosen for LCP transfer functions (which was mentioned above), ensures that LCP uses a strict, finite-height subset of the full CP transfer-functions lattice that is closed under join and function composition operations. The trade-off is that LCP transfer functions for assignment statements whose RHS is not a linear expression and for conditionals are less precise than the corresponding CP transfer functions.

Our final assumption is that procedures other than "main" may send messages, but should not have any "receive" operations. Previous approaches that have addressed data flow analysis or verification problems for asynchronous systems with recursive procedures also have the same restriction [54,29,19].

We now introduce important terminology. The ***demand*** of a given path $p$ in the VCFG is a vector of size $r$, and is defined as follows:

$$demand(p) = \begin{cases} max(\mathbf{0} - w, \mathbf{0}), & \text{if } p = (v \xrightarrow{f,w} z) \\ max(demand(p') - w, \mathbf{0}), & \text{if } p = (e.p'), \text{where } e \equiv (v \xrightarrow{f,w} z) \end{cases}$$

Intuitively, the demand of a path $p$ is the minimum required vector of counter values in any starting configuration at the entry of the path for there to exist a sequence of transitions among configurations that manages to traverse the entire path (following the rule given before Section 2.1). It is easy to see that a path $p$ is feasible *iff* $demand(p) = \mathbf{0}$.

A set of paths $C$ is said to ***cover*** a path $p$ iff: (a) all paths in $C$ have the same start and end nodes (respectively) as $p$, (b) for each $p' \in C$, $demand(p') \leq demand(p)$, and (c) $(\sqcup_{p' \in C} ptf(p')) \sqsupseteq ptf(p)$. (Regarding (b), any binary vector operation in this paper is defined as applying the same operation on every pair of corresponding entries, i.e., point-wise.)

A *path template* $(p_1, p_2, \ldots, p_n)$ of any procedure $F_i$ is a sequence of paths in the VCFG of $F_i$ such that: (a) path $p_1$ begins at the entry node $en_{F_i}$ of $F_i$ and path $p_n$ ends at return node $ex_{F_i}$ of $F_i$, (b) for all $p_i, 1 \leq i < n$, $p_i$ ends at a call-site node, and (c) for all $p_i, 1 < i \leq n$, $p_i$ begins at a return-site node $v_r^i$, such that $v_r^i$ corresponds to the call-site node $v_c^{i-1}$ at which $p_{i-1}$ ends.

#### 3.2 Properties of Demand and Covering

At a high level, Backward DFAS works by growing paths in the backward direction by a single edge at a time starting from the target node (node $k$ in our example in Figure 3). Every time this process results in a path reaching the *start* node (node $a$ in our example), and the path is feasible, the approach simply transfers the entry value $d_0$ via this path to the target node. The main challenge is that due to the presence of cycles and recursion, there are an infinite number of feasible paths in general. In this subsection we present a set of lemmas that embody our intuition on how a finite subset of the set of all paths can be enumerated such that the join of the values brought by these paths is equal to the JOFP. We then present our complete approach in Section 3.3.

***Demand Coverage Lemma:*** *Let* $p_2$ *and* $p_2'$ *be two paths from a node* $v_i$ *to a node* $v_j$ *such that* $demand(p_2') \leq demand(p_2)$*. If* $p_1$ *is any path ending at* $v_i$*, then* $demand(p_1.p_2') \leq demand(p_1.p_2)$*.* □

This lemma can be argued using induction on the length of path $p_1$. A similar observation has been used to solve coverability of lossy channels and well-structured transition systems in general [3,18,2]. An important corollary of this lemma is that for any two paths $p_2'$ and $p_2$ from $v_i$ to $v_j$ such that $demand(p_2') \leq demand(p_2)$, if there exists a path $p_1$ ending at $v_i$ such that $p_1.p_2$ is feasible, then $p_1.p_2'$ is also feasible.

***Function Coverage Lemma:*** *Let $p_2$ be a path from a node $v_i$ to a node $v_j$, and $P_2$ be a set of paths from $v_i$ to $v_j$ such that $(\bigsqcup_{p_2' \in P_2} ptf(p_2')) \sqsupseteq ptf(p_2)$. Let $p_1$ be any path ending at $v_i$ and $p_3$ be any path beginning at $v_j$. Under the distributivity assumption stated in Section 3.1, the following property holds: $(\bigsqcup_{p_2' \in P_2} ptf(p_1.p_2'.p_3)) \sqsupseteq ptf(p_1.p_2.p_3)$.* □

The following result follows from the Demand and Function Coverage Lemmas and from monotonicity of the transfer functions:

***Corollary 1:*** *Let $p_2$ be a path from a node $v_i$ to a node $v_j$, and $P_2$ be a set of paths from $v_i$ to $v_j$ such that $P_2$ covers $p_2$. Let $p_1$ be any path ending at $v_i$. Then, the set of paths $\{p_1.p_2' \mid p_2' \in P_2\}$ covers the path $p_1.p_2$.* □

We now use the running example from Figure 3 to illustrate how we leverage Corollary 1 in our approach. When we grow paths in backward direction from the target node $k$, two candidate paths that would get enumerated (among others) are $p_i \equiv hijk$ and $p_j \equiv hijkhijk$ (in that order). Now, $p_i$ covers $p_j$. Therefore, by Corollary 1, any backward extension $p_1.p_j$ of $p_j$ ($p_1$ is any path prefix) is guaranteed to be covered by the analogous backward extension $p_1.p_i$ of $p_i$. By definition of covering, it follows that $p_1.p_i$ brings in a data value that conservatively over-approximates the value brought in by $p_1.p_j$. Therefore, our approach discards $p_j$ as soon as it gets enumerated. To summarize, our approach discards any path as soon as it is enumerated if it is covered by some subset of the previously enumerated and retained paths.

Due to the finite height of the transfer functions lattice, and because demand vectors cannot contain negative values, at some point in the algorithm every new path that can be generated by backward extension at that point would be discarded immediately. At this point the approach would terminate, and soundness would be guaranteed by definition of covering.

In the inter-procedural setting the situation is more complex. We first present two lemmas that set the stage. The lemmas both crucially make use of the assumption that recursive procedures are not allowed to have "receive" operations. For any path $p_a$ that contains no receive operations, and for any demand vector $d$, we first define $supply(p_a, d)$ as $min(s, d)$, where $s$ is the sum of the queuing vectors of the edges of $p_a$.

***Supply Limit Lemma:*** *Let $p_1, p_2$ be two paths from $v_i$ to $v_j$ such that there are no receive operations in $p_1$ and $p_2$. Let $p_b$ be any path beginning at $v_j$. If $demand(p_b) = d$, and if $supply(p_1, d) \geq supply(p_2, d)$, then $demand(p_1.p_b) \leq demand(p_2.p_b)$.* □

A set of paths $P$ is said to ***d-supply-cover*** a path $p_a$ iff: (a) all paths in $P$ have the same start node and same end node (respectively) as $p_a$, (b) $(\sqcup_{p' \in P} ptf(p')) \sqsupseteq ptf(p_a)$, and (c) for each $p' \in P$, $supply(p', d) \geq supply(p_a, d)$.

***Supply Coverage Lemma:*** *If $p_a.p_b$ is a path, and $demand(p_b) = d$, and if a set of paths $P$ $d$-supply-covers $p_a$, and $p_a$ as well as all paths in $P$ have no receive operations, then the set of paths $\{p'.p_b \,|\, p' \in P\}$ covers the path $p_a.p_b$.*

*Proof argument:* Since $P$ $d$-supply-covers $p_a$, by the Supply Limit Lemma, we have (a): for all $p' \in P$, $demand(p'.p_b) \leq demand(p_a.p_b)$. Since $P$ $d$-supply-covers $p_a$, we also have $(\sqcup_{p' \in P} ptf(p')) \sqsupseteq ptf(p_a)$. From this, we use the Function Coverage lemma to infer that (b): $(\sqcup_{p' \in P} ptf(p'.p_b)) \sqsupseteq ptf(p_a.p_b)$. The result now follows from (a) and (b). □

Consider path *hijk* in our example, which gets enumerated and retained (as discussed earlier). This path gets extended back as *qhijk*; let us denote this path as $p'$. Let $d$ be the demand of $p'$ (i.e., is equal to 3). Our plan now is to extend this path in the backward direction all the way up to node $p$, by prepending interprocedurally valid and complete (i.e., IVC) paths of procedure `foo` in front of $p'$. An IVC path is one that begins at the entry node of `foo`, ends at the return node of `foo`, is of arbitrary calling depth, has balanced calls and returns, and has no pending returns when it completes [50]. First, we enumerate the IVC path(s) with calling-depth zero (i.e., path *co* in the example), and prepend them in front of $p'$. We then produce deeper IVC paths, in phases. In each phase $i$, $i > 0$, we inline IVC paths of calling-depth $i-1$ that have been enumerated and retained so far into the path templates of the procedure to generate IVC paths of calling-depth $i$, and prepend these IVC paths in front of $p'$. We terminate when each IVC path that is generated in a particular phase $j$ is $d$-supply-covered by some subset $P$ of IVC paths generated in previous phases.

The soundness of discarding the IVC paths of phase $j$ follows from the Supply Coverage lemma ($p'$ would take the place of $p_b$ in the lemma's statement, while the path generated in phase $j$ would take the place of $p_a$ in the lemma statement). The termination condition is guaranteed to be reached eventually, because: (a) the supplies of all IVC paths generated are limited to $d$, and (b) the lattice of transfer functions is of finite height. Intuitively, we could devise a sound termination condition even though deeper and deeper IVC paths can increment counters more and more, because a deeper IVC path that increments the counters beyond the demand of $p'$ does not really result in lower overall demand when prepended before $p'$ than a shallower IVC path that also happens to meet the demand of $p'$ (Supply Limit lemma formalizes this).

In our running example, for the path *qhijk*, whose demand is equal to three, prefix generation for it happens to terminate in the fifth phase. The IVC paths that get generated in the five phases are, respectively, $p_0 = co$, $p_1 = cdefgmcono$, $p_2 = (cdefgm)^2 co(no)^2$, $p_3 = (cdefgm)^3 co(no)^3$, $p_4 = (cdefgm)^4 co(no)^4$, and $p_5 = (cdefgm)^5 co(no)^5$. $supply(p_3, 3) = supply(p_4, 3) = supply(p_5, 3) = 3$. The LCP transfer functions of the paths are as follows. $ptf(p_3)$ is (t'=1, x'=x+3, y'=x+2, z'=1), $ptf(p_4)$ is (t'=1, x'=x+4, y'=x+3, z'=1), while $ptf(p_5)$ is (t'=1, x'=x+5, y'=x+4, z'=1). $\{p_3, p_4\}$ 3-supply-covers $p_5$.

We also need a result that when the IVC paths in the $j$th phase are $d$-supply-covered by paths generated in preceding phases, then the IVC paths that would be generated in the $(j+1)th$ would also be $d$-supply-covered by paths generated

**Algorithm 1** Backward DFAS algorithm

**procedure** ComputeJOFP(*target*)
  ▷ Returns JOFP from *start* $\in$ *Nodes* to *target* $\in$ *Nodes*, entry value $d_0 \in \mathcal{L}$.
  **for all** $v \in$ *Nodes* **do**  ▷ *Nodes* is the set of all nodes in the VCFG
    $sPaths(v) = \emptyset$
  For each intra-proc VCFG edge $v \rightarrow target$, add this edge to *workList* and to $sPaths(v)$
  **repeat**
    Remove any path $p$ from *workList*.
    Let $v_1$ be the start node of $p$.
    **if** $v_1$ is a return-site node, with incoming return edge from func. $F_1$ **then**
      Let $v_3$ be the call-site node corresponding to $v_1$, $e_1$ be the call-site-to-entry edge from $v_3$ to $en_{F_1}$, and $r_1$ be the exit-to-return-site edge from $ex_{F_1}$ to $v_1$.
      **for all** $p_1 \in$ ComputeEndToEnd($F_1$, $demand(p)$) **do**
        $p_2 = e_1.p_1.r_1.p$
        **if** Covered($p_2$, $sPaths(v_3)$) returns *false* **then**
          Add $p_2$ to $sPaths(v_3)$ and to *workList*.
    **else if** $v_1$ is the entry node of a func. $F_1$ **then**
      **for all** $v_3 \in$ *call-sites* $(F_1)$ **do**
        Let $e_1$ be the call edge from $v_3$ to $v_1$.
        $p_2 = e_1.p$.
        **if** Covered($p_2$, $sPaths(v_3)$) returns *false* **then**
          Add $p_2$ to $sPaths(v_3)$ and to *workList*.
    **else**
      **for all** intra-procedural edges $e = v_3 \xrightarrow{f,w} v_1$ in the VCFG **do**
        **if** Covered($e.p$, $sPaths(v_3)$) returns *false* **then**
          Add the path $(e.p)$ to $sPaths(v_3)$ and to *workList*.
  **until** *workList* is empty
  $P = \{p \mid p \in sPaths(start), demand(p) = \overline{0}\}$
  **return** $\bigsqcup_{p \in P} (ptf(p))(d_0)$

in phases that preceded $j$. This can be shown using a variant of the Supply Coverage Lemma, which we omit in the interest of space. Once this is shown, it then follows inductively that none of the phases after phase $j$ are required, which would imply that it would be safe to terminate.

The arguments presented above were in a restricted setting, namely, that there is only one call in each procedure, and that only recursive calls are allowed. These restrictions were assumed only for simplicity, and are not actually assumed in the algorithm to be presented below.

#### 3.3 Data Flow Analysis Algorithm

Our approach is summarized in Algorithm 1. ComputeJOFP is the main routine. The algorithm works on a given iVCFG (which is an implicit parameter to the algorithm), and is given a *target* node at which the JOFP is to be computed.

**Algorithm 2** Routines invoked for inter-procedural processing in Backward DFAS algorithm

**procedure** ComputeEndToEnd($F$, $d$)
 ▷ Returns a set of paths that $d$-supply-covers each IVC path of the procedure $F$.
  **for all** $F_i \in \mathit{Funcs}$ **do**
    Place all 0-depth paths from $F_i$ in $\mathit{sIVCPaths}(F_i, d)$
  **repeat**
    pathsAdded = *false*
    **for all** path template $(p_1, p_2, \ldots, p_n)$ in any function $F_i \in \mathit{Funcs}$ **do**
      Let $F_1$ be the procedure called from the call-site at which $p_1$ ends, $F_2$ be the procedure called from the call-site at which $p_2$ ends, and so on.
      **for all** $p'_1 \in \mathit{sIVCPaths}(F_1, d)$, $p'_2 \in \mathit{sIVCPaths}(F_2, d), \ldots$ **do**
        Let $p' = p_1.e_1.p'_1.r_1.p_2.e_2.p'_2.r_2.\ldots.p_n$, where each $e_i$ is the call-edge that leaves the call-site node at which $p_i$ ends and $r_i$ is the return edge corresponding to $e_i$.
        **if** DSCovered($p', d, \mathit{sIVCPaths}(F_i, d)$) returns *false* **then**
          Add the path $p'$ to $\mathit{sIVCPaths}(F_i, d)$. pathsAdded = *true*.
  **until** pathsAdded is *false*
  **return** $\mathit{sIVCPaths}(F, d)$

A key data structure in the algorithm is *sPaths*; for any node $v$, $\mathit{sPaths}(v)$ is the set of all paths that start from $v$ and end at *target* that the algorithm has generated and retained so far. The *workList* at any point stores a subset of the paths in *sPaths*, and these are the paths of the iVCFG that need to be extended backward.

To begin with, all edges incident onto *target* are generated and added to the sets *sPaths* and *workList* (Line 4 in Algorithm 1). In each step the algorithm picks up a path $p$ from *workList* (Line 6), and extends this path in the backward direction. The backward extension has three cases based on the start node of the path $p$. The simplest case is the intra-procedural case, wherein the path is extended backwards in all possible ways by a single edge (Lines 21-23). The routine Covered, whose definition is not shown in the algorithm, checks if its first argument (a path) is covered by its second argument (a set of paths). Note, covered paths are not retained.

When the start node of $p$ is the entry node of a procedure $F_1$ (Lines 14-19), the path is extended backwards via all possible call-site-to-entry edges for procedure $F_1$.

If the starting node of path $p$ is a return-site node $v_1$ (Lines 8-13) in a calling procedure, we invoke a routine ComputeEndToEnd (in line 10 of Algorithm 1). This routine, which we explain later, returns a set IVC paths of the called procedure such that *every* IVC path of the called procedure is $d$-supply-covered by some subset of paths in the returned set, where $d$ denotes $\mathit{demand}(p)$. These returned IVC paths are prepended before $p$ (Line 11), with the call-edge $e_1$ and return edge $r_1$ appropriately inserted.

The final result returned by the algorithm (see Lines 25 and 26 in Algorithm 1) is the join of the values transferred by the zero-demand paths (i.e., feasible paths) starting from the given entry value $d_0 \in \mathcal{L}$.

*Routine* COMPUTEENDTOEND*:* This routine is specified in Algorithm 2, and is basically a generalization of the approach that we described in Section 3.2, now handling multiple call-sites in each procedure, mutual recursion, calls to non-recursive procedures, etc. We do assume for simplicity of presentation that there are no cycles (i.e., loops) in the procedures, as this results in a fixed number of path templates in each procedure. There is no loss of generality here because we allow recursion. The routine incrementally populates a group of sets – there is a set named $sIVCPaths(F_i, d)$ for each procedure $F_i$ in the system. The idea is that when the routine completes, $sIVCPaths(F_i, d)$ will contain a set of IVC paths of $F_i$ that $d$-supply-cover all IVC paths of $F_i$. Note that we simultaneously populate covering sets for all the procedures in the system in order to handle mutual recursion.

The routine COMPUTEENDTOEND first enumerates and saves all zero-depth paths in all procedures (see Line 3 in Algorithm 2). The routine then iteratively takes a path template at a time, and fills in the "holes" between corresponding (call-site, return-site) pairs of the form $v_c^{i-1}, v_r^i$ in the path template with IVC paths of the procedure that is called from this pair of nodes, thus generating a deeper IVC path (see the loop in lines 6-11). A newly generated IVC path $p'$ is retained only if it is not $d$-supply-covered by other IVC paths already generated for the current procedure $F_i$ (Lines 10-11). The routine terminates when no more IVC paths that can be retained are generated, and returns the set $sIVCPaths(F, d)$.

#### 3.4 Illustration

We now illustrate our approach using the example in Figure 3. Algorithm 1 would start from the target node $k$, and would grow paths one edge at a time. After four steps the path $hijk$ would be added to $sPaths(h)$ (the intermediate steps would add suffixes of this path to $sPaths(i)$, $sPaths(j)$, and $sPaths(k)$). Next, path $khijk$ would be generated and discarded, because it is covered by the "root" path $k$. Hence, further iterations of the cycle are avoided. On the other hand, the path $hijk$ would get extended back to node $q$, resulting in path $qhijk$ being retained in $sPaths(q)$. This path would trigger a call to routine COMPUTEENDTOEND. As discussed in Section 3.2, this routine would return the following set of paths: $p_0 = co$, and $p_i = (cdefgm)^i co(no)^i$ for each $1 \leq i \leq 4$. (Recall, as discussed in Section 3.2, that $(cdefgm)^5 co(no)^5$ and deeper IVC paths are 3-supply-covered by the paths $\{p_3, p_4\}$.)

Each of the paths returned above by the routine COMPUTEENDTOEND would be prepended in front of $qhijk$, with the corresponding call and return edges inserted appropriately. These paths would then be extended back to node $a$. Hence, the final set of paths in $sPaths(a)$ would be $abpcoqhijk$, $abpcdefgmconoqhijk$, $abp(cdefgm)^2 co(no)^2$, $abp(cdefgm)^3 co(no)^3$, and $abp(cdefgm)^4 co(no)^4$. Of these paths, the first two are ignored, as they are not feasible. The initial data-flow value (in which all variables are non-constant) is sent via the remaining three paths. In all these three paths the final values of variables 't' and 'z' are one. Hence, these two constants are inferred at node $k$.

#### 3.5 Properties of the algorithm

We provide argument sketches here about the key properties of Backward DFAS. Detailed proofs are available in the appendix that accompanies this paper [4].

*Termination.* The argument is by contradiction. For the algorithm to not terminate, one of the following two scenarios must happen. The first is that an infinite sequence of paths gets added to some set $sPaths(v)$. By Higman's lemma it follows that embedded within this infinite sequence there is an infinite sequence $p_1, p_2, \ldots$, such that for all $i$, $demand(p_i) \leq demand(p_{i+1})$. Because the algorithm never adds covered paths, it follows that for all $i$: $\bigsqcup_{1 \leq k \leq i+1} ptf(p_k) \sqsupset \bigsqcup_{1 \leq k \leq i} ptf(p_k)$. However, this contradicts the assumption that the lattice of transfer functions is of finite height. The second scenario is that an infinite sequence of IVC paths gets added to some set $sIVCPaths(F, d)$ for some procedure $F$ and some demand vector $d$ in some call to routine ComputeEndToEnd. Because the "supply" values of the IVC paths are bounded by $d$, it follows that embedded within the infinite sequence just mentioned there must exist an infinite sequence of paths $p_1, p_2, \ldots$, such that for all $i$, $supply(p_i, d) \geq supply(p_{i+1}, d)$. However, since $d$-supply-covered paths are never added, it follows that for all $i$: $\bigsqcup_{1 \leq k \leq i+1} ptf(p_k) \sqsupset \bigsqcup_{1 \leq k \leq i} ptf(p_k)$. However, this contradicts the assumption that the lattice of transfer functions is of finite height.

*Soundness and Precision.* We already argued informally in Section 3.2 that the algorithm explores all feasible paths in the system, omitting only paths that are covered by other already-retained paths. By definition of covering, this is sufficient to guarantee over-approximation of the JOFP. The converse direction, namely, under-approximation, is obvious to see as every path along which the data flow value $d_0$ is sent at the end of the algorithm is a feasible path. Together, these two results imply that the algorithm is guaranteed to compute the precise JOFP.

*Complexity.* We show the complexity of our approach in the single-procedure setting. Our analysis follows along the lines of the analysis of the backwards algorithm for coverability in VASS [6]. The overall idea, is to use the technique of Rackoff [48] to derive a bound on the length of the paths that need to be considered. We derive a complexity bound of $O(\Delta . h^2 . \mathbf{L}^{2r+1} . r . log(\mathbf{L}))$, where $\Delta$ is the total number of transitions in the VCFG, $Q$ is the number of VCFG nodes, $h$ is the height of lattice of $\mathcal{L} \to \mathcal{L}$ functions, and $\mathbf{L} = (Q.(h+1).2)^{(3r)!+1}$.

## 4 Forward DFAS Approach

The Backward DFAS approach, though precise, requires the transfer function lattice to be of finite height. Due to this restriction, infinite-height abstract domains like Octagons [44], which need *widening* [12], are not accommodated by Backward DFAS. To address this, we present the Forward DFAS approach, which admits *any* complete lattice as an abstract domain (if the lattice is of infinite height then a widening operator should also be provided). The trade-off is precision. Forward DFAS elides only some of the infeasible paths in the VCFG, and hence, in general, computes a conservative *over-approximation* of the JOFP. Forward DFAS is conceptually not as sophisticated as Backward DFAS, but is still a novel proposal from the perspective of the literature.

The Forward DFAS approach is structured as an instantiation of Kildall's data flow analysis framework [32]. This framework needs a given complete lattice, the elements of which will be propagated around the VCFG as part of the fix point computation. Let $\mathcal{L}$ be the given underlying finite or infinite complete lattice. $\mathcal{L}$ either needs to not have any infinite *ascending chains* (e.g., Constant Propagation), or $\mathcal{L}$ needs to have an associated widening operator "$\nabla_{\mathcal{L}}$". The complete lattice $D$ that we use in our instantiation of Kildall's framework is defined as $D \equiv D_{r,\kappa} \rightarrow \mathcal{L}$, where $\kappa \geq 0$ is a user-given non-negative integer, and $D_{r,\kappa}$ is the set of all vectors of size $r$ (where $r$ is the number of counters in the VCFG) such that all entries of the vectors are integers in the range $[0, \kappa]$. The ordering on this lattice is as follows: $(d_1 \in D) \sqsubseteq (d_2 \in D)$ iff $\forall c \in D_{r,\kappa}.\ d_1(c) \sqsubseteq_{\mathcal{L}} d_2(c)$. If a widening operator $\nabla_{\mathcal{L}}$ has been provided for $\mathcal{L}$, we define a widening operator $\nabla$ for $D$ as follows: $d_1 \nabla d_2 \ \equiv\ \lambda c \in D_{r,\kappa}.\ d_1(c) \nabla_{\mathcal{L}} d_2(c)$.

We now need to define the abstract transfer functions with signature $D \rightarrow D$ for the VCFG edges, to be used within the data flow analysis. As an intermediate step to this end, we define a ternary relation *boundedMove1* as follows. Any triple of integers $(p, q, s) \in \mathit{boundedMove1}$ iff

$$\begin{aligned}
&(0 \leq p \leq \kappa) \wedge \\
&((q \geq 0 \wedge p + q \leq \kappa \wedge s = p + q) \vee && (a)\\
&\ (q \geq 0 \wedge p + q > \kappa \wedge s = \kappa) \vee && (b)\\
&\ (q < 0 \wedge p = \kappa \wedge 0 \leq s \leq \kappa \wedge \kappa - s \leq -1 * q) \vee && (c)\\
&\ (q < 0 \wedge p < \kappa \wedge p + q \geq 0 \wedge s = p + q)) && (d)
\end{aligned}$$

We now define a ternary relation *boundedMove* on vectors. A triple of vectors $(c_1, c_2, c_3)$ belongs to relation *boundedMove* iff all three vectors are of the same size, and for each index $i$, $(c_1[i], c_2[i], c_3[i]) \in \mathit{boundedMove1}$.

We now define the $D \rightarrow D$ transfer function for the VCFG edge $q_1 \xrightarrow{f,w} q_2$ as follows:

$$\mathit{fun}(l \in D)\ \equiv\ \lambda c_2 \in D_{r,\kappa}.\ \left( \bigsqcup_{c_1 \text{ such that } (c_1, w, c_2) \in \mathit{boundedMove}} f(l(c_1)) \right)$$

Finally, let $l_0$ denote following function: $\lambda c \in D_{r,\kappa}.$ if $c$ is $\mathbf{0}$ then $d_0$ else $\bot$, where $d_0 \in \mathcal{L}$. We can now invoke Kildall's algorithm using the *fun* transfer functions defined above at all VCFG edges, using $l_0$ as the fact at the "entry" to the "main" procedure. After Kildall's algorithm has finished computing the fix point solution, if $l_v^D \in D$ is the fix point solution at any node $v$, we return the value $\left(\sqcup_{c \in D_{r,\kappa}} l_v^D(c)\right)$ as the final result at $v$.

The intuition behind the approach above is as follows. If $v$ is a vector in the set $D_{r,\kappa}$, and if $(c, m)$ is a channel-message pair, then the value in the $(c, m)$th slot of $v$ encodes the number of instances of message $m$ in channel $c$ currently. An important note is that if this value is $\kappa$, it actually indicates that there are $\kappa$ or more instances of message $m$ in channel $c$, whereas if the value is less than $\kappa$ it represents itself. Hence, we can refer to vectors in $D_{r,\kappa}$ as *bounded queue configurations*. If $d \in D$ is a data flow fact that holds at a node of the VCFG after data flow analysis terminates, then for any $v \in D_{r,\kappa}$ if $d(v) = l$, it indicates that $l$ is a (conservative over-approximation) of the join of the data flow facts brought by all feasible paths that reach the node such that the counter values at the ends of these paths are as indicated by $v$ (the notion of what counter values are indicated by a vector $v \in D_{r,\kappa}$ was described earlier in this paragraph).

The relation *boundedMove* is responsible for blocking the propagation along some of the infeasible paths. The intuition behind it is as follows. Let us consider a VCFG edge $q_1 \xrightarrow{f:\mathcal{L}\to\mathcal{L},w} q_2$. If $c_1$ is a bounded queue configuration at node $q_1$, then, $c_1$ upon propagation via this edge will become a bounded queue configuration $c_2$ at $q_2$ iff $(c_1, w, c_2) \in$ *boundedMove*. Lines $(a)$ and $(b)$ in the definition of *boundedMove1* correspond to sending a message; line $(b)$ basically throws away the precise count when the number of messages in the channel goes above $\kappa$. Line $(c)$ corresponds to receiving a message when all we know is that the number of messages currently in the channel is greater than or equal to $\kappa$. Line $(d)$ is key for precision when the channel has less than $\kappa$ messages, as it allows a receive operation to proceed only if the requisite number of messages are present in the channel.

The formulation above extends naturally to inter-procedural VCFGs using generic inter-procedural frameworks such as the *call strings* approach [55]. We omit the details of this in the interest of space.

***Properties of the approach:*** Since Forward DFAS is an instantiation of Kildall's algorithm, it derives its properties from the same. As the set $D_{r,k}$ is a finite set, it is easy to see that the fix-point algorithm will terminate.

To argue the soundness of the algorithm, we consider the *concrete lattice* $D_c \equiv D_r \to \mathcal{L}$, and the following "concrete" transfer function for the VCFG edge $q_1 \xrightarrow{f,w} q_2$: $fun\_conc(l \in D_c) \equiv \lambda c_2 \in D_r. \left(\bigsqcup_{c_1 \in D_r \text{ such that } c_1+w=c_2} f(l(c_1))\right)$, where $D_r$ is the set of all vectors of size $r$ of natural numbers. We then argue that the abstract transfer function *fun* defined earlier is a *consistent abstraction* [12] of *fun_conc*. This soundness argument is given in detail in the appendix that accompanies this paper [4].

If we restrict our discussion to single-procedure systems, the complexity of our approach is just the complexity of applying Kildall's algorithm. This works out to $O(Q^2\kappa^r h)$, where $Q$ is the number of VCFG nodes, and $h$ is either the height of the lattice $\mathcal{L}$ or the maximum increasing sequence of values from $\mathcal{L}$

that is obtainable at any point using the lattice $\mathcal{L}$ in conjunction with Kildall's algorithm, using the given widening operation $\nabla_{\mathcal{L}}$.

| $c$ | t | x | y | z |
|---|---|---|---|---|
| 1 | 0 | 0 | 0 | 0 |

(1)

| $m$ | t | x | y | z |
|---|---|---|---|---|
| 2 | 0 | 1 | 0 | 1 |

(2)

| $c$ | t | x | y | z |
|---|---|---|---|---|
| 1 | 0 | 0 | 0 | 0 |
| 2 | 0 | 1 | 0 | 1 |

(3)

| $m$ | t | x | y | z |
|---|---|---|---|---|
| 2 | 0 | 1 | 0 | 1 |
| 3 | 1 | 2 | 1 | 1 |

(4)

| $c$ | t | x | y | z |
|---|---|---|---|---|
| 1 | 0 | 0 | 0 | 0 |
| 2 | 0 | 1 | 0 | 1 |
| 3 | 1 | 2 | 1 | 1 |

(5)

| $m$ | t | x | y | z |
|---|---|---|---|---|
| 2 | 0 | 1 | 0 | 1 |
| 3 | 1 | $\top$ | $\top$ | 1 |

(6)

| $o$ | t | x | y | z |
|---|---|---|---|---|
| 1 | 0 | 0 | 0 | 0 |
| 2 | 0 | 1 | 0 | 1 |
| 3 | 1 | $\top$ | $\top$ | 1 |

(7)

| $k$ | t | x | y | z |
|---|---|---|---|---|
| 1 | 1 | $\top$ | $\top$ | 1 |
| 2 | 1 | $\top$ | $\top$ | 1 |
| 3 | 1 | $\top$ | $\top$ | 1 |

(8)

**Fig. 4.** Data flow facts over a run of the algorithm

***Illustration:*** We illustrate Forward DFAS using the example in Figure 3. Figure 4 depicts the data flow values at four selected nodes as they get updated over eight selected points of time during the run of the algorithm. In this illustration we assume a context insensitive analysis for simplicity (it so happens that context sensitivity does not matter in this specific example). We use the value $\kappa = 3$. Each small table is a data flow fact, i.e., an element of $D \equiv D_{r,\kappa} \rightarrow \mathcal{L}$. The top-left cell in the table shows the node at which the fact arises. In each row the first column shows the counter value, while the remaining columns depict the known constant value of the variables ($\top$ indicates unknown). Here are some interesting things to note. When any tuple of constant values transfers along the path from node $c$ to node $m$, the constant values get updated due to the assignment statements encountered, *and* this tuple shifts from counter $i$ to counter $i+1$ (if $i$ is not already equal to $\kappa$) due to the "send" operation encountered. When we transition from Step (5) to Step (6) in the figure, we get $\top$'s, as counter values 2 and 3 in Step (5) both map to counter value 3 in Step (6) due to $\kappa$ being 3 (hence, the constant values get *joined*). The value at node $o$ (in Step (7)) is the join of values from Steps (5) and (6). Finally, when the value at node $o$ propagates to node $k$, the tuple of constants associated with counter value 3 end up getting mapped to all lower values as well due to the receive operations encountered.

Note, the precision of our approach in general increases with the value of $\kappa$ (the running time increases as well). For instance, if $\kappa$ is set to 2 (rather than 3) in the example, some more infeasible paths would be traversed. Only $\mathrm{z} = 1$ would be inferred at node $k$, instead of ($\mathrm{t} = 1$, $\mathrm{z} = 1$).

### 5 Implementation and Evaluation

We have implemented prototypes of both the Forward DFAS and Backward DFAS approaches, in Java. Both the implementations have been parallelized, using the ThreadPool library. With Backward DFAS the iterations of the outer "repeat" loop in Algorithm 1 run in parallel, while with Forward DFAS propagations of values from different nodes to their respective successors happen in parallel. Our implementations currently target systems without procedure calls, as none of our benchmarks had recursive procedure calls.

Our implementations accept a given system, and a "target" control state $q$ in one of the processes of the system at which the JOFP is desired. They then construct the VCFG from the system (see Section 2.1), and identify the *target set* of $q$, which is the set of VCFG nodes in which $q$ is a constituent. For instance, in Figure 2, the target set for control state $e$ is $\{(a, e), (b, e)\}$. The JOFPs at the nodes in the target set are then computed, and the join of these JOFPs is returned as the result for $q$.

Each variable reference in any transition leaving any control state is called a "use". For instance, in Figure 2, the reference to variable x along the outgoing transition from state $d$ is one use. In all our experiments, the objective is to find the uses that are definitely constants by computing the JOFP at all uses. This is a common objective in many research papers, as finding constants enables optimizations such as constant folding, and also checking assertions in the code. We instantiate Forward DFAS with the Constant Propagation (CP) analysis, and Backward DFAS with the LCP analysis (for the reason discussed in Section 3.1). We use the bound $\kappa = 2$ in all runs of Forward DFAS, except with two benchmarks which are too large to scale to this bound. We discuss this later in this section. All the experiments were run on a machine with 128GB RAM and four AMD Opteron 6386 SE processors (64 cores total).

#### 5.1 Benchmarks and modeling

**Table 1.** Information about the benchmarks. Abbreviations used: (a) prtcl = protocol, (b) comm = communication, (c) app = application

| **Benchmark (1)** | **Description (2)** | **#Proc (3)** | **#Var (4)** | $r$ **(5)** | **#VCFG nodes (6)** |
|---|---|---|---|---|---|
| mutex | mutual exclusion example | 3 | 1 | 6 | 4536 |
| bartlett | Bartlett's alternating-bit prtcl | 3 | 3 | 7 | 17864 |
| leader | leader election prtcl | 2 | 11 | 12 | 16002 |
| lynch | distorted channel comm prtcl | 3 | 5 | 27 | 168912 |
| peterson | Peterson's mutual exclusion prtcl | 3 | 4 | 4 | 6864 |
| boundedAsync | illustrative example | 3 | 5 | 10 | 14375 |
| receive1 | illustrative example | 2 | 5 | 13 | 1160 |
| server | actor-based client server app | 3 | 3 | 6 | 1232 |
| chameneos | Chameneos concurrency game | 3 | 9 | 10 | 45584 |
| replicatingStorage | replicating storage system | 4 | 4 | 8 | 47952 |
| event_bus_test | publish-subscribe system | 2 | 2 | 5 | 160 |
| jobqueue_test | concurrent job queue system | 4 | 1 | 10 | 28800 |
| bookCollectionStore | REST app | 2 | 2 | 12 | 2162 |
| nursery_test | structured concurrency app | 3 | 2 | 4 | 1260 |

We use 14 benchmarks for our evaluations. These are described in the first two columns of Table 1. Four benchmarks – bartlett, leader, lynch, and peterson – are Promela models for the Spin model-checker. Three benchmarks – boundedAsync, receive1, and replicatingStorage – are from the P language repository (www.github.com/p-org). Two benchmarks – server and chameneos – are from the Basset repository (www.github.com/SoftwareEngineeringToolDemos/FSE-2010-Basset). Four benchmarks – event_bus_test, jobqueue_test, nursery_test, and bookCollectionStore – are real world Go programs. There is one toy example "mutex", for ensuring mutual exclusion, via blocking receive messages, that we have made ourselves. We provide precise links to the benchmarks in the appendix [4].

Our DFAS implementations expect the asynchronous system to be specified in an XML format. We have developed a custom XML schema for this, closely based on the Promela modeling language used in Spin [26]. We followed this direction in order to be able to evaluate our approach on examples from different languages. We manually translated each benchmark into an XML file, which we call a *model*. As the input XML schema is close to Promela, the Spin models were easily translated. Other benchmarks had to be translated to our XML schema by understanding their semantics.

Note that both our approaches are expensive in the worst-case (exponential or worse in the number of counters $r$). Therefore, we have chosen benchmarks that are moderate in their complexity metrics. Still, these benchmarks are real and contain complex logic (e.g., the leader election example from Promela, which was discussed in detail in Section 1.1). We have also performed some manual simplifications to the benchmarks to aid scalability (discussed below). Our evaluation is aimed towards understanding the impact on precision due to infeasible paths in real benchmarks, and not necessarily to evaluate applicability of our approach to large systems.

We now list some of the simplifications referred to above. Language-specific idioms that were irrelevant to the core logic of the benchmark were removed. The number of instances of identical processes in some of the models were reduced in a behavior-preserving manner according to our best judgment. In many of the benchmarks, messages carry *payload*. Usually the payload is one byte. We would have needed 256 counters just to encode the payload of one 1-byte message. Therefore, in the interest of keeping the analysis time manageable, the payload size was reduced to 1 bit or 2 bits. The reduction was done while preserving key behavioral aspects according to our best judgment. Finally, procedure calls were inlined (there was no use of recursion in the benchmarks).

In the rest of this section, whenever we say "benchmark", we actually mean the model we created corresponding to the benchmark. Table 1 also shows various metrics of our benchmarks (based on the XML models). Column 3-6 depict, respectively, the number of processes, the total number of variables, the number of "counters" $r$, and the total number of nodes in the VCFG. We provide our XML models of all our benchmarks, as well as full output files from the runs of our approach, as a downloadable folder (https://drive.google.com/drive/folders/181DloNfm6_UHFyz7qni8rZjwCp-a8oCV).

#### 5.2 Data flow analysis results

**Table 2.** Data flow analysis results

| Benchmark (1) | #Var. uses (2) | #Asserts (3) | DFAS Approach | | | | Baseline Approaches | | | |
|---|---|---|---|---|---|---|---|---|---|---|
| | | | #Consts. (4) | | #Verified (5) | | #Consts. (6) | | #Verified (7) | |
| | | | Forw. | Back. | Forw. | Back. | JOP | CCP | JOP | CCP |
| mutex | 6 | 2 | 6 | 6 | 2 | 2 | 0 | 0 | 0 | 0 |
| bartlett | 9 | 1 | 0 | 0 | 0 | 0 | 0 | 0 | 0 | 0 |
| leader | 54 | 4 | 20 | 6 | 4 | 0 | 6 | 6 | 2 | 0 |
| lynch | 6 | 2 | 4 | 3 | 0 | 0 | 4 | 3 | 0 | 0 |
| peterson | 14 | 2 | 0 | 0 | 0 | 0 | 0 | 0 | 0 | 0 |
| boundedAsync | 24 | 8 | 8 | 8 | 0 | 0 | 8 | 8 | 0 | 0 |
| receive1 | 9 | 5 | 8 | 8 | 4 | 4 | 2 | 8 | 2 | 4 |
| server | 4 | 1 | 0 | 0 | 0 | 0 | 0 | 0 | 0 | 0 |
| chameneos | 35 | 2 | 2 | 2 | 0 | 0 | 2 | 2 | 0 | 0 |
| replicatingStorage | 8 | 1 | 2 | 0 | 1 | 0 | 0 | 0 | 0 | 0 |
| event_bus_test | 5 | 3 | 3 | 3 | 3 | 3 | 0 | 2 | 0 | 2 |
| jobqueue_test | 3 | 1 | 0 | 1 | 0 | 1 | 0 | 0 | 0 | 0 |
| bookCollectionStore | 10 | 8 | 8 | 10 | 6 | 8 | 0 | 8 | 0 | 6 |
| nursery_test | 2 | 2 | 2 | 2 | 2 | 2 | 0 | 2 | 0 | 2 |
| **Total** | 189 | 42 | 63 | 49 | 22 | 20 | 22 | 39 | 4 | 14 |

We structure our evaluation as a set of research questions (RQs) below. Table 2 summarizes results for the first three RQs, while Table 3 summarizes results for RQ 4.

***RQ 1:*** *How many constants are identified by the Forward and Backward DFAS approaches?* Column (2) in Table 2 shows the number of *uses* in each benchmark. Columns (4)-Forw and (4)-Back show the number of uses identified as constants by the Forward and Backward DFAS approaches, respectively. In total across all benchmarks Forward DFAS identifies 63 constants whereas Backward DFAS identifies 49 constants.

Although in aggregate Backward DFAS appears weaker than Forward DFAS, Backward DFAS infers more constants than Forward DFAS in two benchmarks – jobqueue_test and bookCollectionStore. Therefore, the two approaches are actually incomparable. The advantage of Forward DFAS is that it can use relatively more precise analyses like CP that do not satisfy the assumptions of Backward DFAS, while the advantage of Backward DFAS is that it always computes the precise JOFP.

***RQ 2:*** *How many assertions are verified by the approaches?* Verifying assertions that occur in code is a useful activity as it gives confidence to developers. All but one of our benchmarks had assertions (in the original code itself, before modeling).

We carried over these assertions into our models. For instance, for the benchmark *leader*, the assertion appears in Line 11 in Figure 1. In some benchmarks, like jobqueue_test, the assertions were part of test cases. It makes sense to verify these assertions as well, as unlike in testing, our technique considers all possible interleavings of the processes. As "bookCollectionStore" did not come with any assertions, a graduate student who was unfamiliar with our work studied the benchmark and suggested assertions.

Column (3) in Table 2 shows the number of assertions present in each benchmark. Columns (5)-Forw and (5)-Back in Table 2 show the number of assertions declared as safe (i.e., verified) by the Forward and Backward DFAS approaches, respectively. An assertion is considered verified iff constants (as opposed to "$\top$") are inferred for all the variables used in the assertion, and if these constants satisfy the assertion. As can be seen from the last row in Table 2, both approaches verify a substantial percentage of all the assertions – 52% by Forward DFAS and 48% by Backward DFAS. We believe these results are surprisingly useful, given that our technique needs no loop invariants or usage of theorem provers.

***RQ 3:*** *Are the DFAS approaches more precise than baseline approaches?* We compare the DFAS results with two baseline approaches. The first baseline is a Join-Over-all-Paths (JOP) analysis, which basically performs CP analysis on the VCFG without eliding any infeasible paths. Columns (6)-JOP and (7)-JOP in Table 2 show the number of constants inferred and the number of assertions verified by the JOP baseline. It can be seen that Backward DFAS identifies 2.2 times the number of constants as JOP, while Forward DFAS identifies 2.9 times the number of constants as JOP (see columns (4)-Forw, (4)-Back, and (6)-JOP in the **Total** row in Table 2). In terms of assertions, each of them verifies almost 5 times as many assertions as JOP (see columns (5)-Forw, (5)-Back, and (7)-JOP in **Total** row in Table 2.) It is clear from the results that eliding infeasible paths is extremely important for precision.

The second baseline is Copy Constant Propagation (CCP) [50]. This is another variant of constant propagation that is even less precise than LCP. However, it is based on a finite lattice, specifically, an IFDS [50] lattice. Hence this baseline represents the capability of the closest related work to ours [29], which elides infeasible paths but supports only IFDS lattices, which are a sub-class of finite lattices. (Their implementation also used a finite lattice of predicates, but we are not aware of a predicate-identification tool that would work on our benchmarks out of the box.) We implemented the CCP baseline within our Backward DFAS framework. This baseline hence computes the JOFP using CCP (i.e., it elides infeasible paths).

Columns (6)-CCP and (7)-CCP in Table 2 show the number of constants inferred and the number of assertions verified by the CCP baseline. From the **Total** row in Table 2 it can be seen that Forward DFAS finds 62% more constants than CCP, while Backward DFAS finds 26% more constants than CCP. With respect to number of assertions verified, the respective gains are 57% and 43%. In other words, infinite domains such as CP or LCP can give significantly more precision than closely related finite domains such as CCP.

**Table 3.** Execution time in seconds

| | mut. | bar. | lea. | lyn. | pet. | bou. | rec. | ser. | cha. | rep. | eve. | job. | boo. | nur. |
|---|---|---|---|---|---|---|---|---|---|---|---|---|---|---|
| Forw | 1.2 | 14.0 | 1.3 | 8.0 | 1.2 | 21.0 | 1.2 | 1.2 | 18.0 | 2.4 | 1.2 | 1.2 | 1.2 | 1.2 |
| Back | 5.0 | 11.0 | 284.0 | 118.0 | 13.0 | 21.0 | 8.0 | 3.0 | 220.0 | 21.0 | 3.0 | 140.0 | 16.0 | 1.0 |
| JOP | 1.2 | 1.3 | 1.6 | 8.0 | 1.2 | 1.4 | 1.3 | 1.2 | 3.1 | 3.0 | 1.1 | 1.4 | 1.2 | 1.2 |
| CCP | 5.0 | 12.0 | 226.0 | 116.0 | 12.0 | 14.0 | 8.0 | 3.0 | 156.0 | 24.0 | 3.0 | 51.0 | 30.0 | 1.0 |

***RQ 4:*** *How does the execution cost of DFAS approaches compare to the cost of the JOP baseline?* The columns in Table 3 correspond to the benchmarks (only first three letters of each benchmark's name are shown in the interest of space). The rows show the running times for Forward DFAS, Backward DFAS, JOP baseline, and CCP baseline, respectively.

The JOP baseline was quite fast on almost all benchmarks (except lynch). This is because it maintains just a single data flow fact per VCFG node, in contrast to our approaches. Forward DFAS was generally quite efficient, except on chameneos and lynch. On these two benchmarks, it scaled only with $\kappa = 1$ and $\kappa = 0$, respectively, encountering memory-related crashes at higher values of $\kappa$ (we used $\kappa = 2$ for all other benchmarks). These two benchmarks have large number of nodes and a high value of $r$, which increases the size of the data flow facts.

The running time of Backward DFAS is substantially higher than the JOP baseline. One reason for this is that being a demand-driven approach, the approach is invoked separately for each *use* (Table 2, Col. 2), and the cumulative time across all these invocations is reported in the table. In fact, the mean time per query for Backward DFAS is less than the total time for Forward DFAS on 9 out of 14 benchmarks, in some cases by a factor of 20x. Also, unlike Forward DFAS, Backward DFAS visits a small portion of the VCFG in each invocation. Therefore, Backward DFAS is more memory efficient and scales to all our benchmarks. Every invocation of Backward DFAS consumed less than 32GB of memory, whereas with Forward DFAS, three benchmarks (leader, replicatingStorage, and jobqueue_test) required more than 32GB, and two (lynch and chameneos) needed more than the 128 GB that was available in the machine. On the whole, the time requirement of Backward DFAS is still acceptable considering the large precision gain over the JOP baseline.

#### 5.3 Limitations and Threats to Validity

The results of the evaluation using our prototype implementation are very encouraging, in terms of both usefulness and efficiency. The evaluation does however pose some threats to the validity of our results. The benchmark set, though extracted from a wide set of sources, may not be exhaustive in its idioms. Also, while modeling, we had to simplify some of the features of the benchmarks in order to let the approaches scale. Therefore, applicability of our approach directly on real systems with all their language-level complexities, use of libraries, etc., is not yet established, and would be a very interesting line of future work.

### 6 Related Work

The modeling and analysis of *parallel systems*, which include asynchronous systems, multi-threaded systems, distributed systems, event-driven systems, etc., has been the focus of a large body of work, for a very long time. We discuss some of the more closely related previous work, by dividing the work into four broad categories.

*Data Flow Analysis:* The work of Jhala et al. [29] is the closest work that addresses similar challenges as our work. They combine the Expand, Enlarge and Check (EEC) algorithm [21] that answers control state reachability in WSTS [18], with the unordered channel abstraction, and the IFDS [50] algorithm for data flow analysis, to compute the JOFP solution for all nodes. They admit only IDFS abstract domains, which are finite by definition. Some recent work has extended this approach for analyzing JavaScript [60] and Android [45] programs. Both our approaches are dissimilar to theirs, and we admit infinite lattices (like CP and LCP). On the other hand, their approach is able to handle parameter passing between procedures, which we do not.

Bronevetsky et al. [8] address generalized data flow analysis of a very restricted class of systems, where any receive operation must receive messages from a specific process, and channel contents are not allowed to cause non-determinism in control flow. Other work has addressed analysis of asynchrony in web applications [28,42]. These approaches are efficient, but over-approximate the JOFP by eliding only certain specific types of infeasible paths.

*Formal Modeling and Verification:* Verification of asynchronous systems has received a lot of attention over a long time. VASS [31] and Petri nets [49] (which both support unordered channel abstraction) have been used widely to model parallel and asynchronous processes [31,38,54,29,19,5]. Different analysis problems based on these models have been studied, such as reachability of configurations [7,43,34,35], coverability and boundedness [31,3,2,18,21,6], and coverability in the presence of stacks or other data structures [57,5,9,10,40].

The *coverability* problem mentioned above is considered equivalent to control state reachability, and has received wide attention [1,14,29,19,54,20,33,5,56]. Abdulla et al. [3] were the first to provide a backward algorithm to answer coverability. Our Backward DFAS approach is structurally similar to their approach, but is a strict generalization, as we incorporate data flow analysis using infinite abstract domains. (It is noteworthy that when the abstract domain is finite, then data flow analysis can be reduced to coverability.) One difference is that we use the unordered channel abstraction, while they use the lossy channel abstraction. It is possible to modify our approach to use lossy channels as well (when there are no procedure calls, which they also do not allow); we omit the formalization of this due to lack of space.

Bouajjani and Emmi [5] generalize over previous coverability results by solving the coverability problem for a class of multi-procedure systems called recursively parallel programs. Their class of systems is somewhat broader than ours, as they allow a caller to receive the messages sent by its callees. Our ComputeEndToEnd routine in Algorithm 2 is structurally similar to their approach. They admit finite abstract domains only. It would be interesting future work to extend the Backward DFAS approach to their class of systems.

Our approaches explore all interleavings between the processes, following the Spin semantics. Whereas, the closest previous approaches [29,5] only address "event-based" systems, wherein a set of processes execute sequentially without interleaving at the statement level, but over an unbounded schedule (i.e., each process executes from start to finish whenever it is scheduled).

*Other forms of verification:* Proof-based techniques have been explored for verifying asynchronous and distributed systems [24,58,47,22]. These techniques need inductive variants and are not as user-friendly as data flow analysis techniques. Behavioral types have been used to tackle specific analysis problems such as deadlock detection and correct usage of channels [36,37,52].

*Testing and Model Checking:* Languages and tools such as Spin and Promela [26], P [15], P# [13], and JPF-Actor [39] have been used widely to model-check asynchronous systems. A lot of work has been done in testing of asynchronous systems [16,13,53,23,59] as well. Such techniques are bounded in nature and cannot provide the strong verification guarantees that data flow analysis provides.

### 7 Conclusions and Future Work

In spite of the substantial body of work on analysis and verification of distributed systems, there is no existing approach that performs precise data flow analysis of such systems using infinite abstract domains, which are otherwise very commonly used with sequential programs. We propose two data flow analysis approaches that solve this problem – one computes the precise JOFP solution always, while the other one admits a fully general class of infinite abstract domains. We have implemented our approaches, analyzed 14 benchmarks using the implementation, and have observed substantially higher precision from our approach over two different baseline approaches.

Our approach can be extended in many ways. One interesting extension would be to make Backward DFAS work with infinite height lattices, using widening. Another possible extension could be the handling of parameters in procedure calls. There is significant scope for improving the scalability using better engineering, especially for Forward DFAS. One could explore the integration of partial-order reduction [11] into both our approaches. Finally, we would like to build tools based on our approach that apply directly to programs written in commonly-used languages for distributed programming.
